# The Role of a Novel Gene, *GmXTH-like26*, in Improving Salt Tolerance in Soybean

**DOI:** 10.3390/plants15131948

**Published:** 2026-06-24

**Authors:** Tongyu Cheng, Dan Yao, Zhou Sun, Zhuo Zhang, Sujie Fan, Qi Zhang, Min Xu, Songnan Yang, Yang Song, Jun Zhang

**Affiliations:** School of College of Agronomy, Jilin Agricultural University, 2888 Xincheng Street, Nanguan District, Changchun 130118, China; m13931862432@163.com (T.C.); danyaojlau@163.com (D.Y.); 15373114317@163.com (Z.S.); z175882124@163.com (Z.Z.); sujiefan527939862@163.com (S.F.); zhangxiaoqi6969@163.com (Q.Z.); minxu1771097192@163.com (M.X.); soy@jlau.edu.cn (S.Y.)

**Keywords:** soybean, salt tolerance, *GmXTH-like26*, cell wall, physiological response

## Abstract

Soybean is an important crop for food, oil and feed production in China, and improving its yield is a major national goal. Salt stress severely restricts soybean production. XTH genes participate in plant growth and stress adaptation, yet the functions of most soybean XTH members are unclear. In this study, we cloned the soybean *GmXTH-like26* gene previously identified via transcriptome sequencing, and successfully constructed its overexpression vector and CRISPR/Cas9 gene-editing vector. Subcellular localization analysis confirmed that *GmXTH-like26* is localized to the cell wall. The gene was transformed into soybean via the Agrobacterium-mediated method. Under 100 mM NaCl stress, the *GmXTH-like26*-overexpressing lines exhibited markedly enhanced salt tolerance at both germination and seedling stages compared with the control group. Physiological and biochemical assays showed that the overexpression plants had higher activities of superoxide dismutase (SOD), peroxidase (POD) and catalase (CAT), lower malondialdehyde (MDA) content and higher chlorophyll content under salt stress, while the gene-edited lines displayed the opposite trends. These results indicate that *GmXTH-like26* improves salt tolerance in soybean by reducing reactive oxygen species accumulation and effectively enhances the resistance of soybean to salt stress.

## 1. Introduction

Soil salinization represents a global environmental challenge that restricts agricultural productivity, impairs ecosystem stability, and hinders sustainable agricultural development [1,2]. The extent of soil salinization is expanding globally, driven by climate warming, declining precipitation, and unsustainable irrigation management [3]. According to the Food and Agriculture Organization (FAO), by 2024, approximately 1.38 billion hectares of land were affected by salinity and alkalinity, including one-third of all irrigated areas, significantly reducing crop yields [4]. Saline soils impair water uptake by plant roots and severely restrict growth and reproduction, ultimately lowering crop productivity [5,6,7]. In soybean, salt stress disrupts normal growth and development via multiple physiological and cellular pathways, including ionic imbalance, osmotic stress, oxidative damage, and elevated pH [8,9,10]. The seedling stage is particularly vulnerable to salt stress, as salinity suppresses seed germination rates and inhibits root growth [11,12]. As roots play a critical role in water and nutrient uptake, impaired root development compromises whole-plant growth, hinders the formation of vegetative and reproductive organs, and ultimately reduces yield potential [13]. Consistently, studies by Md Shawquat et al. have shown that soybean yield declines progressively as soil salinity increases [14]. Salt stress also induces massive accumulation of reactive oxygen species (ROS), which triggers membrane lipid peroxidation, impairs the stability of cell membrane systems and exacerbates oxidative damage. Along with the duration of stress, these changes further alter the accumulation and partitioning of organic compounds in plants and inhibit vegetative growth processes such as root elongation and plant height increase in seedlings [15]. Therefore, plants enhance salt tolerance by regulating ion transport, accumulating osmoregulatory substances and activating the antioxidant enzyme system to adapt to adverse environments. This can alleviate the damage caused by salt–alkali stress to soybean germplasm resources and provide a theoretical basis for breeding new salt-tolerant soybean varieties.

The XTH (*xyloglucan endotransglucosylase/hydrolase*) gene family is widely present across plant species and plays critical roles in growth, development, and responses to abiotic stress [16,17,18,19]. XTH enzymes regulate the elasticity and extensibility of the primary cell wall, thereby influencing plant stress tolerance.

Across species, XTH genes exhibit diverse responses to stresses such as drought, salinity, metal toxicity, heat, and cold. In soybean, overexpression of *GmXTH1* enhances seedling tolerance to salt and drought stress by promoting root development, strengthening antioxidant defenses, maintaining ion balance, and preserving leaf hydration and photosynthetic efficiency [20,21]. Similarly, in Arabidopsis, *XTH11*, *XTH29*, and *XTH33* contribute to drought stress responses [22], while wheat XTH genes regulate drought tolerance throughout plant development [23]. In various plant species, XTH genes have been shown to respond to diverse abiotic stresses. For instance, XTH genes in poplar [24], Arabidopsis [25], and grapevine [26] are transcriptionally induced by salt stress. In ramie, *BnXTH1* has been demonstrated to enhance cadmium tolerance [27]; in banana, *MaXTH* is involved in cold stress responses [28]; and in rapeseed, *BnXTH* family members are associated with aluminum tolerance [29]. Additionally, *LsXTH43* improves lettuce seed germination under heat stress [30], and *XTH19* enhances cold tolerance in Arabidopsis [31]. Beyond abiotic stress, heterologous expression of *GmXTH43* in cotton increases resistance to nematode infection [32]. These findings collectively highlight the essential role of XTH genes in both biotic and abiotic stress responses [33]. Therefore, the identification and functional characterization of XTH family members are critical for advancing our understanding of salt tolerance in soybean and for providing a theoretical foundation for developing novel salt-tolerant cultivars.

## 2. Results

### 2.1. Vector Construction and Validation of the Soybean GmXTH-like26 Gene

#### 2.1.1. Cloning of the *GmXTH-like26* Gene

Total RNA was extracted from the soybean cultivar M18 and quantified using a nucleic acid-protein analyzer, yielding a concentration of 1178 ng/μL. RNA integrity was confirmed via agarose gel electrophoresis, which showed distinct 28S, 18S, and 5S rRNA bands at the expected positions (Figure S1A), indicating that the RNA was suitable for downstream experiments.

The RNA was reverse-transcribed into cDNA, which served as a template for PCR amplification of the *GmXTH-like26* gene. The PCR product appeared as a single band of the expected size (Figure S1B), confirming successful amplification. The target fragment was excised, purified, and inserted into the pMD-18T vector, which was then transformed into competent *Escherichia coli.* Sequencing of the resulting colonies confirmed that the inserted fragment matched the expected *GmXTH-like26* sequence (Figure S1C), verifying the successful cloning of the gene into the vector.

#### 2.1.2. Construction and Validation of pCAMBIA3301-*GmXTH-like26*

The pCAMBIA3301-*GmXTH-like26* plasmid was verified using double restriction enzyme digestion. Agarose gel electrophoresis revealed two bands corresponding to the expected sizes of the vector and the target gene fragment (Figure S2A). Sequence alignment further confirmed that the inserted coding sequence (CDS) of *GmXTH-like26* matched the expected sequence with 100% identity (Figure S2B), demonstrating the successful construction of the overexpression vector.

### 2.2. Genetic Transformation and Molecular Characterization of GmXTH-like26

#### 2.2.1. Genetic Transformation of *GmXTH-like26* in Glycine Max

The *GmXTH-like26* gene was introduced into the soybean variety JN18 using both overexpression and gene-editing constructs through Agrobacterium-mediated transformation. Transgenic plants were obtained following standard germination and selection procedures. The overall transformation workflow is summarized in Figure S3.

#### 2.2.2. PCR Analysis of T1 Transgenic Plants

Genomic DNA was extracted from the leaves of T1-generation soybean lines to confirm the presence of the transgene. PCR analysis (Figure S4) revealed that four plants showed successful overexpression of *GmXTH-like26*, while two plants were positive for the CRISPR/Cas9 edits.

#### 2.2.3. PCR Analysis of T2 Transgenic Plants

Genomic DNA from T2-generation transgenic soybean plants was analyzed via PCR to assess the presence of the introduced constructs. The analysis identified nine plants with *GmXTH-like26* overexpression and six plants carrying CRISPR/Cas9 edits. Representative gel electrophoresis results are shown in Figure S5, with well labels corresponding to those in Figure S4.

#### 2.2.4. Bar Protein Analysis in T2 Transgenic Plants

Bar test strips were used to detect the Bar protein in T2-generation transgenic soybean plants, allowing identification of positive lines. The results (Figure S6) showed detectable bands in both control (C-line) and test (T-line) plants, confirming expression of the exogenous Bar gene, production of Bar protein, and stable inheritance of the trait. Specifically, samples 1 and 2 were positive for *GmXTH-like26* overexpression, while sample 3 carried the expression vector. These findings indicate that the vector is stably expressed and successfully transmitted to the next generation.

#### 2.2.5. Analysis of Target Site Mutations

The *GmXTH-like26* gene in T2-generation gene-edited soybean plants was amplified via PCR and sequenced to assess mutations at the target locus. All six transgenic plants exhibited mutations, with the types summarized in Figure S7. Specifically, the KO1 mutation involved base substitutions and deletions, KO2 included base deletions and insertions, and KO3 consisted solely of base substitutions.

### 2.3. Bioinformatics Analysis of GmXTH-like26

**Figure 1:**
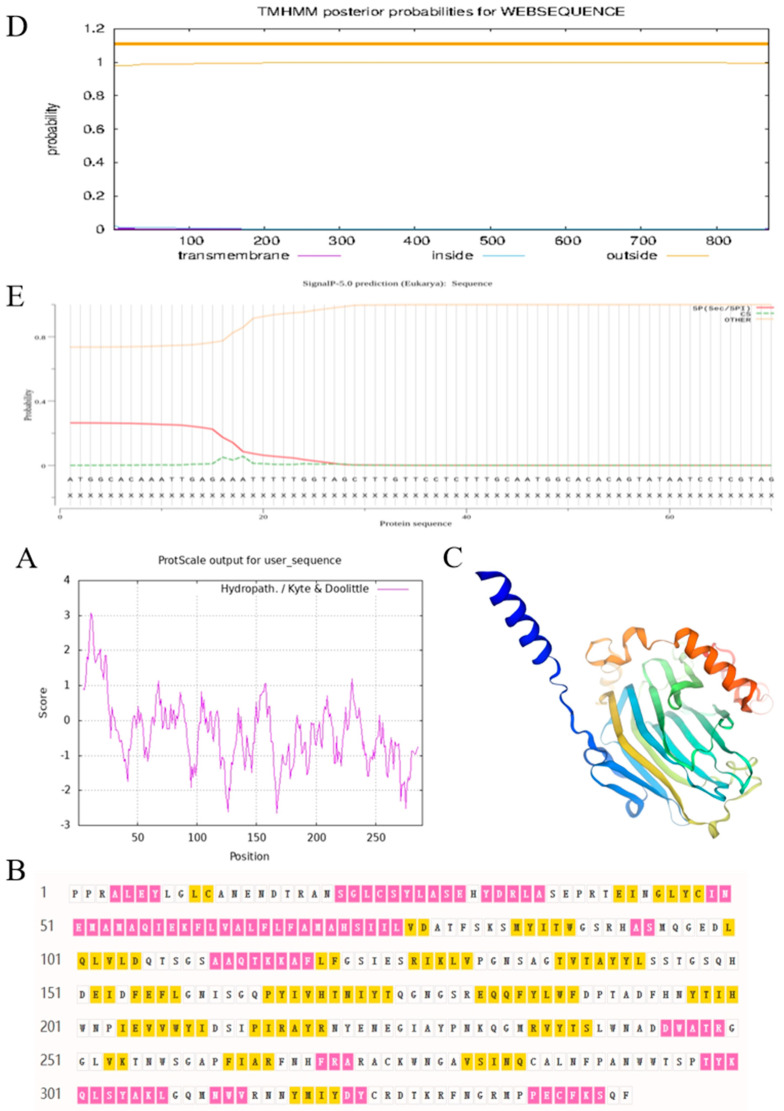
(**A**) Hydrophobicity Prediction of the Amino Acid Sequence Encoded by *GmXTH-like26*; (**B**) Secondary structure analysis of the amino acid sequence encoded by *GmXTH-like26*; (**C**) Tertiary structure analysis of the amino acid sequence encoded by *GmXTH-like26*; (**D**) Analysis of the transmembrane structure of *GmXTH-like26* protein; (**E**) Analysis of *GmXTH-like26* protein signal peptide.

The predicted properties of the *GmXTH-like26* protein were analyzed using the Pfam database (http://pfam.xfam.org/). As summarized in Table 1, the protein comprises 289 amino acids, with a molecular formula of C_1514_H_2237_N_403_O_425_S_12_, a molecular weight of 33,268.58 Da, and a theoretical isoelectric point of 8.76. Hydrophilicity analysis (Figure 1A) indicates that the protein contains more hydrophilic than hydrophobic regions, with an average GRAVY score of −0.343, classifying it as hydrophilic. Secondary structure prediction using ExPASy (http://web.expasy.org/cgi-bin/protscale/protscale.pl (accessed on 22 April 2025)) showed that the protein is predominantly α-helical (Figure 1B). Three-dimensional homology modeling further confirmed the presence of multiple helical structures, consistent with the secondary structure results (Figure 1C). Transmembrane domain analysis (Figure 1D) revealed that *GmXTH-like26* lacks transmembrane regions, indicating it is not a membrane-bound protein. Signal peptide prediction (Figure 1E) showed no detectable signal peptide, classifying it as a non-secreted protein.

### 2.4. Expression Profile of GmXTH-like26 in Soybean

**Figure 2:**
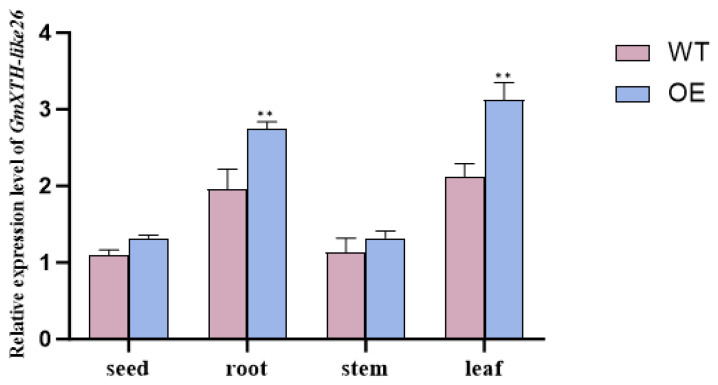
Spatial expression of the *GmXTH-like26* gene (** *p* < 0.01). (WT represents the control group of soybean cultivar JN18, and OE indicates plants overexpressing the *GmXTH-like26* gene. Roots, stems and leaves were sampled from soybean plants at the V1 growth stage).

The spatiotemporal expression of *GmXTH-like26* was quantified in T2-generation soybean plants. The gene exhibited the highest expression in leaves (3.12 ± 0.13) and roots (2.74 ± 0.06), while lower levels were observed in stems (1.32 ± 0.05) and germinating seeds (1.32 ± 0.02). Expression in leaves and roots was significantly higher than in the control plants (Figure 2), indicating tissue-specific regulation of *GmXTH-like26*.

**Figure 3:**
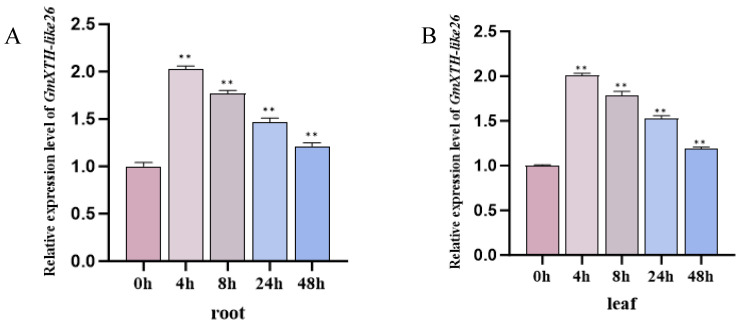
Expression of the *GmXTH-like26* gene under stress conditions (** *p* < 0.01). (**A**) shows the expression of *GmXTH-like26* gene in roots of positive plants; (**B**) shows the expression of *GmXTH-like26* gene in leaves of positive plants. (Total RNA was extracted from roots and leaves of *GmXTH-like26*-overexpressing soybean lines under 100 mM NaCl stress, followed by reverse transcription into cDNA. The expression levels of *GmXTH-like26* were detected at 0 h, 4 h, 8 h, 24 h and 48 h of stress treatment with three biological replicates. The β-actin gene was used as the internal reference).

The expression of *GmXTH-like26* was analyzed in soybean roots exposed to 100 mM NaCl over a time course. Transcript levels were 1.23 ± 0.02 at 0 h, 2.58 ± 0.07 at 4 h, 2.32 ± 0.02 at 8 h, 2.03 ± 0.02 at 24 h, and 1.77 ± 0.02 at 48 h post-treatment. Expression at 4, 8, 24, and 48 h was significantly higher than at 0 h (Figure 3A), indicating that *GmXTH-like26* responds rapidly to salt stress, with transcript levels rising shortly after exposure and gradually declining over time.

The expression of *GmXTH-like26* was measured in soybean leaf tissues under 100 mM NaCl treatment at 0, 4, 8, 24, and 48 h. Transcript levels were 1.19 ± 0.01, 2.46 ± 0.04, 2.17 ± 0.05, 1.78 ± 0.03, and 1.53 ± 0.02, respectively. Compared with the baseline at 0 h, significant upregulation was observed at all subsequent time points (Figure 3B). These results indicate that *GmXTH-like26* responds rapidly to salt stress, with expression peaking early and gradually declining over time. These results indicate that *GmXTH-like26* responds rapidly to salt stress, with expression peaking early and gradually declining over time. The induction of *GmXTH-like26* under saline conditions suggests a potential role in enhancing salt tolerance in soybean.

### 2.5. Subcellular Localization of GmXTH-like26

**Figure 4:**
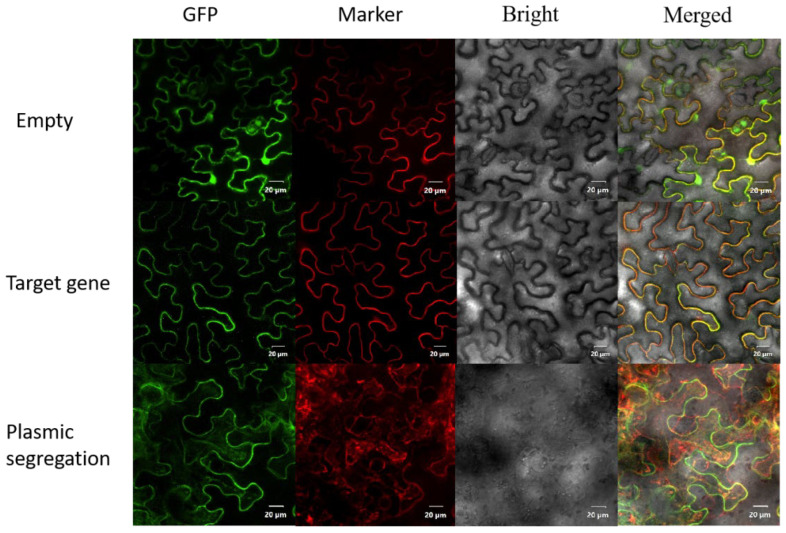
Subcellular localization of *GmXTH-like26* protein in tobacco leaves (Bars = 20 μm, Marker = PMEI1-mCherry).

To determine the subcellular localization of the *GmXTH-like26* protein, a pCAMBIA1302-*GmXTH-like26*-GFP fusion construct was generated and transiently expressed in tobacco leaves via Agrobacterium-mediated transformation, with the empty vector serving as a control. Fluorescence imaging revealed that the *GmXTH-like26*-GFP signal was concentrated at the interface between the cell membrane and the cell wall. Plasmolysis experiments, which separated the cell membrane from the cell wall, confirmed that the fluorescence was restricted to the cell wall (Figure 4). These results indicate that *GmXTH-like26* is localized to the cell wall, suggesting its functional role in cell wall modification.

### 2.6. Functional Analysis of GmXTH-like26 Under Salt Stress

#### 2.6.1. Effects of Salt Stress on Soybean Germination

**Figure 5:**
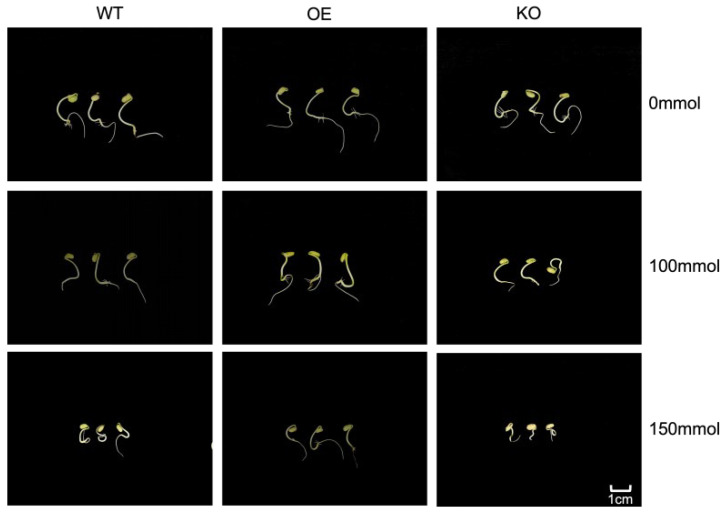
Germination phenotypes of each material transfected with *GmXTH-like26* gene under different treatment conditions (WT represents the JN18 wild-type control. OE and KO are T_2_ generation soybean plants with *GmXTH-like26* overexpression and knockout, respectively. All materials were treated with 0 mM, 100 mM, and 150 mM NaCl for 7 days).

As shown in Figure 5, under control conditions (0 mM NaCl), both wild-type and *GmXTH-like26*-overexpressing plants germinated normally, with no significant differences. Under 100 mM and 150 mM NaCl treatments, overexpressing (OE) plants exhibited significantly longer shoots and higher germination rates compared with the wild type. In contrast, knockout (KO) lines showed reduced germination and shorter shoots under the same conditions. These findings indicate that overexpression of *GmXTH-like26* alleviates the inhibitory effects of salt stress on soybean germination, highlighting its role in enhancing salt tolerance.

Under varying NaCl concentrations, germination rate, germination vigor, and germination index differed among soybean lines expressing *GmXTH-like26* (Table 2). Under non-stress conditions, no significant differences were observed among the lines, except that the KO line exhibited a slightly lower germination rate than the control (note: values reported as 0 are due to rounding; actual values were all > 0 and <0.005). Under 100 mM NaCl, the OE line showed significantly higher germination rate, vigor, and index compared with the control, whereas the KO line displayed the opposite trend. At 150 mM NaCl, the OE line maintained a significantly higher germination rate and germination index relative to the control. These results indicate that *GmXTH-like26* enhances seed germination under salt stress, helping to mitigate the inhibitory effects of salinity on soybean germination.

**Figure 6:**
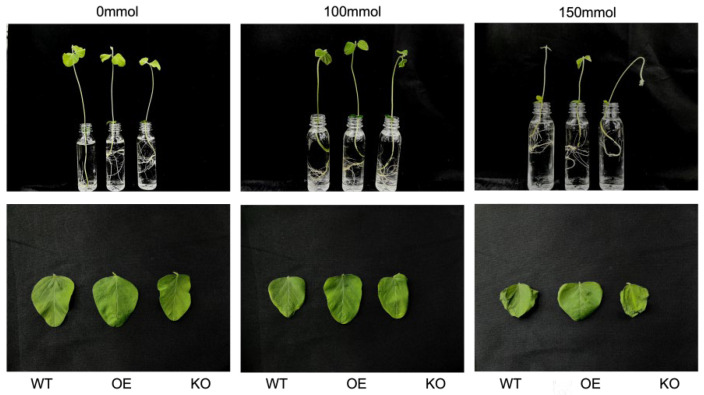
Seedling phenotypes of each material transfected with *GmXTH-like26* gene under different treatment conditions (WT indicates the JN18 wild-type control. OE and KO represent T_2_ generation *GmXTH-like26*-overexpressing and knockout soybean lines, respectively. All seedlings were grown to the V1 stage and then treated with 0 mM, 100 mM, and 150 mM NaCl solutions for 24 h).

As shown in Figure 6, under non-stress conditions, all transgenic and control plants grew vigorously, with no visible wilting or disease symptoms, and no significant differences were observed among the lines. Under 100 mM NaCl stress, the transgenic lines displayed varying salt tolerance. Overexpressing (OE) plants remained vigorous, with erect stems and slightly drooping but normally expanded leaves. In contrast, knockout (KO) plants showed moderate wilting, yellowing and drooping leaves, curved stems, and shriveled foliage, while control plants exhibited mild wilting, slight leaf yellowing, and minor leaf-margin wrinkling. Under 150 mM NaCl stress, OE plants maintained relatively healthy growth, with most stems erect and a portion of leaves normally expanded, although some leaf margins were slightly wrinkled. Control plants displayed severe leaf wrinkling and markedly curved stems, whereas KO plants exhibited extreme leaf curling and stunted, withered stems. These observations indicate that overexpression of *GmXTH-like26* supports vigorous growth, erect stems, and normal leaf expansion under salt stress, thereby enhancing overall salt tolerance in soybean.

#### 2.6.2. Root Phenotypes of *GmXTH-like26*-Expressing Soybean Lines Under Salt Stress

**Figure 7:**
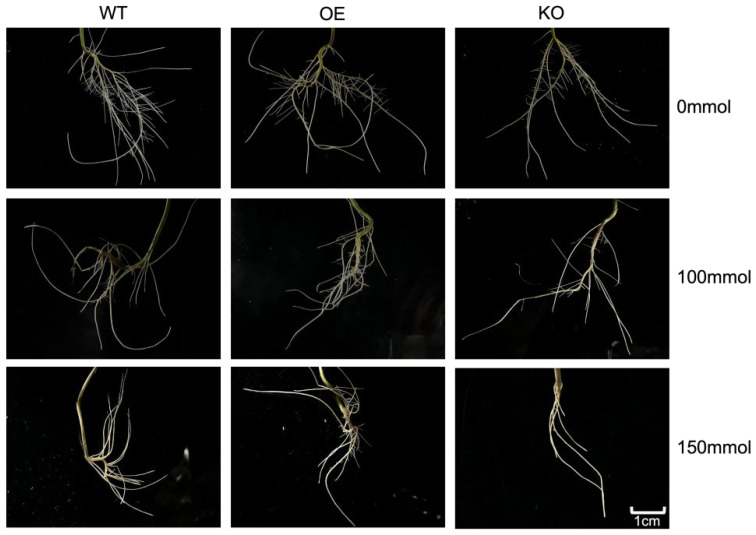
Root phenotypes of each material transform with *GmXTH-like26* gene under different treatment conditions (WT (JN18) served as the control. T_2_ generation *GmXTH-like26*-overexpressing (OE) and knockout (KO) lines were grown to the V1 stage, and treated with 0 mM (control), 100 mM and 150 mM NaCl in plastic bottles (16 cm × 4.6 cm) filled with 200 mL 1/2 Hoagland’s solution. Root phenotypes were measured when plants reached the V2 stage in an artificial climate chamber).

Under non-stress conditions, no significant differences in root growth were observed among the lines; taproots were robust, lateral roots abundant, and numerous fibrous roots were present (Figure 7). Under 100 mM NaCl stress, roots of the overexpression (OE) lines remained relatively healthy. In contrast, the control taproots were slightly curled with a few yellowish-brown spots, and knockout (KO) taproots became thinner, exhibited slower growth, and developed more yellowish-brown lesions. At 150 mM NaCl, OE line roots showed slower growth, reduced lateral root number, and fewer fibrous roots, but overall remained healthier than controls. In the control group, taproots and lateral roots exhibited rot with no new fibrous root formation, while KO roots experienced severe rot and extremely limited growth. These results indicate that overexpression of *GmXTH-like26* enhances root growth and development under salt stress and mitigates salt-induced root damage, highlighting its role in promoting soybean tolerance to salinity.

As shown in Table 3, NaCl stress had a notable impact on root traits, including total root length, root volume, root surface area, and average root diameter, across the different *GmXTH-like26*-expressing lines. Under control conditions, the total root length of overexpression (OE) plants was significantly greater than that of the control group. At 100 mM NaCl, most root traits did not differ significantly between OE plants and the control, except that knockout (KO) plants exhibited a significant reduction in root surface area. Under higher stress (150 mM NaCl), OE plants showed marked increases in total root length, root surface area, and average root diameter compared with the control. In contrast, KO plants displayed significant decreases in total root length, root volume, and root surface area, while other traits remained largely unchanged. These results suggest that *GmXTH-like26* contributes to root growth and development during the seedling stage, enhancing soybean tolerance to salt stress.

#### 2.6.3. *GmXTH-like26* Enhances Antioxidant Enzyme Activity Under Salt Stress

Under non-stress conditions, no significant differences were observed in the activities of superoxide dismutase (SOD), peroxidase (POD), or catalase (CAT) among the *GmXTH-like26*-expressing lines. Similarly, malondialdehyde (MDA) content and total chlorophyll levels were largely comparable, except that knockout (KO) plants showed slightly lower chlorophyll content than the control.

**Figure 8:**
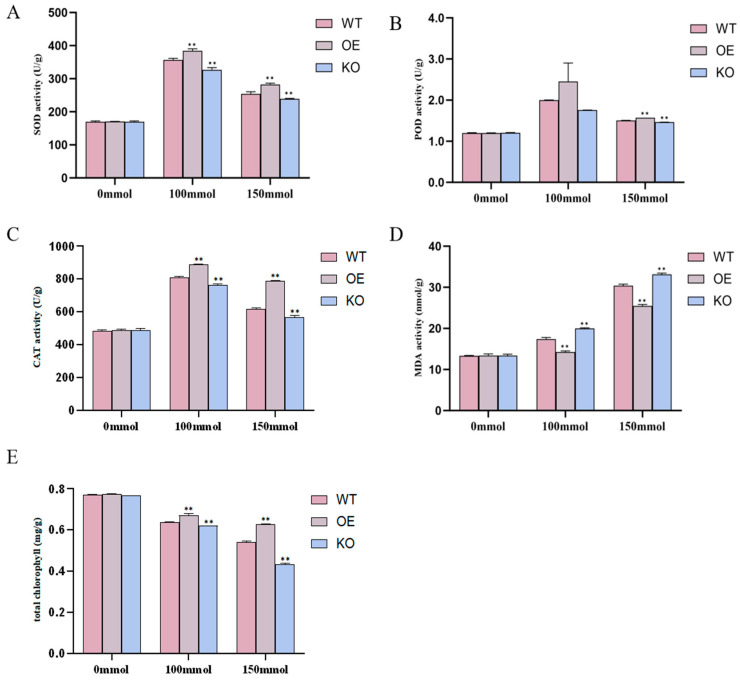
Seedling physiological and biochemical indexes of each material transfected with *GmXTH-like26* gene under different treatment conditions (**A**) SOD activity(U/g FW); (**B**) POD activity(U/g FW); (**C**) CAT activity(U/g FW); (**D**) MDA content(nmol/g FW); (**E**) Total chlorophyll content (mg/g FW). (** *p* < 0.01) (Data are presented as mean ± standard deviation (SD) of three biological replicates).

Under NaCl stress, overexpression (OE) of *GmXTH-like26* significantly enhanced antioxidant enzyme activities. SOD activity in OE plants reached 384.37 U/g and 282.89 U/g under 100 mM and 150 mM NaCl, respectively, compared with 356.80 U/g and 253.85 U/g in the control, while KO plants exhibited lower activity (326.79 U/g and 238.79 U/g) (Figure 8A). POD activity in OE plants was also higher under 150 mM NaCl (1.57 U/g) than in the control (1.51 U/g), whereas KO plants showed reduced activity (1.46 U/g) (Figure 8B). Similarly, CAT activity in OE plants under 100 mM and 150 mM NaCl stress reached 888.70 U/g and 788.69 U/g, respectively, exceeding the control (812.08 U/g and 616.93 U/g), while KO plants exhibited lower CAT activity (764.27 U/g and 568.98 U/g) (Figure 8C). These results demonstrate that *GmXTH-like26* enhances antioxidant defenses to mitigate oxidative damage under salt stress.

Consistent with these effects, OE plants accumulated significantly less MDA than the control under both 100 mM (14.25 nmol/g vs. 17.38 nmol/g) and 150 mM NaCl (25.44 nmol/g vs. 30.38 nmol/g), whereas KO plants accumulated more MDA (19.98 nmol/g and 33.16 nmol/g, respectively) (Figure 8D), indicating that *GmXTH-like26* reduces lipid peroxidation.

In addition, overexpression of *GmXTH-like26* maintained higher total chlorophyll content under salt stress. At 100 mM NaCl, chlorophyll content in OE plants was 0.67 mg/g, compared with 0.64 mg/g in the control and 0.62 mg/g in KO plants. At 150 mM NaCl, OE plants retained 0.63 mg/g of chlorophyll, exceeding the control (0.54 mg/g) and KO plants (0.44 mg/g) (Figure 8E). These findings suggest that *GmXTH-like26* supports chlorophyll synthesis and photosynthetic capacity under salt stress, contributing to overall salt tolerance.

## 3. Discussion

Salt stress is one of the major abiotic stresses during crop growth and development, which causes osmotic imbalance in plants [34] and results in irreversible damage to plant growth. In this study, the soybean *GmXTH-like26* gene was cloned. By comparing phenotypic and physiological differences between transgenic plants overexpressing this target gene and control plants, we preliminarily explored the function of *GmXTH-like26* in the salt stress response network. The findings provide theoretical support and practical references for breeding new salt-tolerant soybean varieties.

Xyloglucan endotransglucosylases/hydrolases (*XETs/XEHs*, collectively *XTHs*) are enzymes that modify xyloglucan and belong to glycoside hydrolase family 16 [35]. They possess two catalytic activities—xyloglucan endotransglucosylase (XET) and xyloglucan endohydrolase (XEH)—which enable them to loosen, synthesize, and remodel cell walls [36]. Numerous XTH genes have been identified as key regulators of cell wall remodeling in woody plants [37]. A total of 61 XTH family members have been identified in soybean. Li Song et al. classified the XTH family into three subfamilies (I, II and III) based on sequence characteristics, and subfamily III was further divided into subgroups IIIA and IIIB. The *GmXTH-like26* gene was assigned to group I/II [38]. Subfamily IIIA exhibits xyloglucan hydrolase (XEH) activity and specifically hydrolyzes the β-1,4 glycosidic bonds of xyloglucan. By contrast, subfamilies IIIB, I and II predominantly possess xyloglucan endotransglycosylase (XET) activity, which enables the cleavage and reconnection of xyloglucan chains [39]. The conserved catalytic motif of XTH proteins is DEIDFEFLG. Threonine or serine residues near the catalytic site can undergo N-glycosylation modification, which is closely associated with enzyme activity. Conserved N-glycosylation sites are absent in subfamily IIIA, whereas such sites exist in subfamilies I/II [16].

Plant cell walls not only serve as physical barriers against environmental stresses but also actively contribute to signal transduction, ion homeostasis, and antioxidant defense [40]. Dynamic modification and reinforcement of cell wall structure are therefore critical for plants to respond to abiotic stresses and maintain overall resilience [41,42]. *XTHs* display spatiotemporal expression patterns and play important roles in plant growth and development [43]. In this study, *GmXTH-like26* expression in roots and leaves was significantly higher than in the control group. Following 100 mM NaCl treatment, its expression increased markedly at 4, 8, 24, and 48 h in both tissues, suggesting that *GmXTH-like26* contributes to soybean responses to salt stress. In Arabidopsis thaliana, *XTH* gene expression is tissue-specific: five genes are expressed in green siliques, two in stems, and at least ten in the root differentiation zone, where they facilitate cell elongation, expansion, and cell wall formation [44,45]. In *Populus tremula* × *P. tremuloides*, *PttXET16A* is involved in secondary vascular tissue development, and in cotton, higher XTH activity during cell elongation correlates with increased fiber length [46]. The *GmXTH-like26* gene cloned in this study belongs to the XTH family and encodes a hydrophilic protein with xyloglucan endotransglycosylase activity. Its secondary structure is dominated by α-helices, and the tertiary structure adopts a typical multi-helical fold. Transmembrane domain and signal peptide analyses revealed that this protein has no transmembrane regions or signal peptides, indicating it is a non-secretory protein. Subcellular localization assays demonstrated that *GmXTH-like26* functions in the cell wall. These findings suggest that the protein undergoes intracellular transport and extracellular localization via the plant non-classical secretory pathway, and subsequently participates in xyloglucan modification and cell wall remodeling in the cell wall. It is speculated that *GmXTH-like26* improves soybean salt tolerance by stabilizing cell wall structure, mitigating cell wall stress and damage, lowering DAMPs release, and inhibiting RBOH-triggered ROS production.

Muhammad Ali et al. systematically elucidated the regulatory mechanisms of ROS/RNS scavenging and their downstream signaling pathways. They confirmed that stress-induced ROS burst aggravates membrane lipid peroxidation and causes massive accumulation of malondialdehyde (MDA), while antioxidant enzymes can effectively alleviate this process [15]. In the present study, under salt stress, the activities of superoxide dismutase (SOD) and peroxidase (POD) were significantly increased in *GmXTH-like26*-overexpressing transgenic lines, accompanied by lower MDA content compared with the control. In contrast, the gene-edited lines exhibited markedly reduced SOD and POD activities and obviously higher MDA levels than the wild type. Collectively, *GmXTH-like26* indirectly upregulates the expression of antioxidant enzymes including SOD, POD and CAT, activates the ROS scavenging system, mitigates oxidative damage, and ultimately improves salt stress tolerance in soybean.

The root system serves as the first line of defense, detecting stress and regulating water and nutrient uptake, which directly influences plant growth and development [47]. Roots also play roles in storing photosynthetic products and contributing to overall stress resilience [48]. Plant root systems are highly plastic and can adapt to abiotic stress by altering root growth traits [49]. Plants with more robust root systems generally exhibit enhanced tolerance to environmental stress. The plant hormone auxin and the expression of auxin-responsive genes are key regulators of root development [50,51]; modulating these genes can influence root architecture and, consequently, plant stress tolerance. Members of the XTH family are critical for enhancing root stress resistance through cell wall modification. For example, *RsXTH25* is strongly induced by lead stress, and its overexpression improves root hair resistance while reducing oxidative damage [52]. Under saline-alkali stress, *SlXTH9*, regulated by *SlCHP16*, promotes root development, with overexpression of *SlCHP16* resulting in higher root growth rates and improved stress tolerance [53]. Similarly, Arabidopsis *XTH19* and *XTH23* are modulated by brassinosteroid signaling to promote lateral root growth under salt stress [54], while the pepper *CaXTH3* gene reinforces mesophyll cell walls in Arabidopsis, enhancing salt tolerance [55]. Muhammad Ali et al. systematically elucidated the auxin-mediated regulatory mechanism under salt stress. Auxin can inhibit primary root growth, promote lateral root proliferation and optimize root architecture, thereby regulating nutrient uptake in plants exposed to salt stress [56]. In this study, compared with the control group, *GmXTH-like26*-overexpressing plants exhibited superior root growth performance, as well as greater total root length, root surface area and root volume under salt stress. The results indicate that this gene facilitates lateral root formation and remodels root architecture, ultimately enhancing salt tolerance in soybean.

## 4. Materials and Methods

### 4.1. Preparation of Experimental Materials

The soybean cultivars M18 and JN18 utilized in this study were provided by the Center for Plant Biotechnology at Jilin Agricultural University. Seeds of uniform size, healthy appearance, and free from insect damage were selected. After thorough washing with distilled water and surface drying, the seeds were germinated and grown in a controlled-environment chamber until the V1 stage.

Primers for this study were designed using Primer 5.0 and synthesized by Sangon Biotech (Shanghai) Co., Ltd., Shanghai, China. (Supplementary Table S1). Total RNA was extracted from leaves of M18 plants at the V1 stage using the Trizol method, and cDNA was generated through reverse transcription. The *GmXTH-like26* gene was cloned using this cDNA as a template. An overexpression vector, pCAMBIA3301-*GmXTH-like26*, containing the Bar gene as a selectable marker, was constructed. A gene-editing vector, pCBSG015-*GmXTH-like26*, was synthesized by Jiangsu Weimi Biotechnology Co., Ltd., Nanjing, China. Both vectors were introduced into Agrobacterium tumefaciens strain EHA105 and subsequently used to transform soybean plants via Agrobacterium-mediated transformation. Positive transgenic plants were identified via PCR and Bar test strips. In total, nine T2-generation overexpression lines and six T2-generation gene-edited lines were successfully obtained.

### 4.2. Bioinformatics Analysis of the GmXTH-like26 Gene

The *GmXTH-like26* gene sequence used in this study was obtained from our previous transcriptome analysis, and the corresponding protein sequence was retrieved from the NCBI database (https://www.ncbi.nlm.nih.gov). The protein’s basic characteristics were analyzed using the Pfam online database (http://pfam.xfam.org/). Hydrophilicity and secondary structure were evaluated with ExPASy ProtScale (http://web.expasy.org/cgi-bin/protscale/protscale.pl (accessed on 22 April 2025)), and three-dimensional structural modeling was performed using SWISS-MODEL (https://swissmodel.expasy.org/ (accessed on 22 April 2025)). Transmembrane domains were predicted using the TMHMM Server (https://services.healthtech.dtu.dk/service.php?TMHMM-2.0 (accessed on 22 April 2025)), while signal peptide properties were analyzed with SignalP-5.0 (https://services.healthtech.dtu.dk/ (accessed on 22 April 2025)). Additional physicochemical parameters, including molecular formula, molecular weight, and theoretical isoelectric point, were determined using ProtParam (https://web.expasy.org/protparam/ (accessed on 22 April 2025)).

### 4.3. RNA Isolation and Real-Time Quantitative PCR Analysis

Soybean plants overexpressing *GmXTH-like26* were set as the experimental group, and the recipient cultivar JN18 served as the control group. All plants were cultivated in 96-well hydroponic boxes with 1/2 Hoagland’s solution under continuous illumination conditions at 25 °C and 65% relative humidity, with a photoperiod of 16 h light/8 h dark. When the seedlings grew to the V1 stage, salt stress was simulated by treatment with 100 mM NaCl solution supplemented with 1/2 Hoagland’s solution. Root and leaf samples were collected at 0, 4, 8, 24, and 48 h after treatment. In addition, samples from roots, stems, leaves, and germinating seeds were collected from both groups. Total RNA was extracted using a standard RNA extraction kit, and cDNA was synthesized using the TransScript^®^ All-in-One First-Strand cDNA Synthesis SuperMix for qPCR (One-Step gDNA Removal) kit. β-actin was used as an internal control. For each sample, three biological replicates and three technical replicates were performed. Relative expression levels of *GmXTH-like26* were calculated using the 2^−ΔΔCt^ method.

### 4.4. Subcellular Localization Analysis

To determine the subcellular localization of *GmXTH-like26*, a fusion expression vector, pCAMBIA1302-*GmXTH-like26*-GFP, was constructed, with an empty vector serving as a control. The vector was introduced into Agrobacterium tumefaciens strain GV3101, and the transformed Agrobacterium was infiltrated into tobacco leaves. The plants were kept in darkness for 12 h, followed by incubation under low light for 34–48 h. Subcellular localization of *GmXTH-like26* was then observed using a confocal microscope [57].

### 4.5. Analysis of Salt Tolerance in Soybeans Expressing GmXTH-like26

T2-generation soybean plants overexpressing *GmXTH-like26* (OE), T2-generation gene-edited plants (KO), and the recipient cultivar JN18 (WT) were obtained by screening T1-generation positive plants and propagating subsequent generations. All primers used for PCR verification were synthesized by Changchun Kumei Biotechnology Co., Ltd., Changchun, China.

Each replicate consisted of twenty healthy soybean seeds, free from insect damage. Seeds were surface-sterilized by immersion in 75% ethanol followed by 5% sodium hypochlorite for 120 s, rinsed three times with distilled water, and placed in disposable 9 cm Petri dishes lined with moist filter paper. Experiments were performed in triplicate. Seeds were subjected to treatments of 0 mM NaCl (control), 100 mM NaCl, and 150 mM NaCl. Each treatment received 20 mL of the respective NaCl solution, while control seeds received an equal volume of distilled water. Germination was carried out in a climate-controlled incubator with a 14 h light/10 h dark photoperiod at 25 °C and 70% relative humidity. Germination was defined as radicle emergence of at least 1 mm. Germination parameters, including germination rate (GR), germination energy (GE), and germination index (GI), were subsequently calculated.

GR(%) = total number of germinations on the 6th day × 100/number of tested seeds

GE(%) = number of germinations in the first 3 days × 100/number of tested seeds

GI = ∑(DG/DT)

DG is the number of germinations per day.

DT is the number of days corresponding to DG.

### 4.6. Root Phenotypes of GmXTH-like26 Under Salt Stress

Three uniformly vigorous plants were selected from each soybean line. Cultivation and salt stress treatments were conducted as described in Section 4.5. Plants were subjected to three NaCl treatments: 0 mM (control), 100 mM, and 150 mM. Each plant was grown in a plastic bottle (16 cm height × 4.6 cm diameter) filled with 200 mL of 1/2 Hoagland’s nutrient solution. Cultivation was carried out in an artificial climate chamber until the plants reached the V2 growth stage, at which point samples were harvested for root phenotypic analysis. The total root length, root volume, root surface area, and average root diameter were then quantified using a root scanning system.

### 4.7. Evaluation of Physiological and Biochemical Parameters

Plant cultivation and salt stress treatments were performed as described in Section 2.5, with three biological replicates per group. Samples were collected after seven days of growth in the climate-controlled chamber. The activities of superoxide dismutase (SOD, Cat. No. BC0170, nitroblue tetrazolium (NBT) photoreduction method), peroxidase (POD, Cat. No. BC0090, guaiacol method), catalase (CAT, Cat. No. BC0200, UV spectrophotometric method), the content of malondialdehyde (MDA, Cat. No. BC0020, TBA method), and chlorophyll (Cat. No. BC0990, acetone-ethanol mixed solution extraction-visible spectrophotometric method) in plants were determined according to the corresponding instructions of the physiological and biochemical assay kits from Solarbio (Beijing, China) [11].

### 4.8. Data Analysis

All results are presented as the mean ± standard deviation of three replicates. Statistical analyses were performed using SPSS (SPSS 25.0), with significance defined as *p* < 0.05 and high significance as *p* < 0.01. Graphs were prepared using Origin (Origin 2022) software.

## 5. Conclusions

The soybean *GmXTH-like26* gene encodes a 289-amino-acid protein with a molecular weight of 33,268.58 Da. Bioinformatics analysis indicates that it is a hydrophilic, non-secreted protein, and subcellular localization confirmed its presence in the cell wall. *GmXTH-like26* is expressed across multiple soybean tissues, with the highest levels observed in leaves.

Under NaCl stress, plants overexpressing *GmXTH-like26* showed superior germination and seedling growth compared with the control. Overexpression enhanced germination rate, vigor, and seedling index, increased the activities of antioxidant enzymes (SOD, POD, and CAT), reduced the accumulation of the lipid peroxidation product MDA, promoted chlorophyll synthesis, and stimulated root development. Quantitative real-time PCR analysis further demonstrated that *GmXTH-like26* expression is upregulated in response to salt stress.

Overall, these results indicate that *GmXTH-like26* plays a key role in the soybean salt stress response and contributes to enhanced salt tolerance through the regulation of antioxidant defense, chlorophyll content, and root growth.

## Figures and Tables

**Table 1:** Physical and Chemical Properties of *GmXTH-like26* Genes analysis.

Gene Name	Molecular Formula	Protein Size (aa)	Molecular Weight (Da)	Theoretical pI	Aliphatic Index
*GmXTH-like26*	C_1514_H_2237_N_403_O_425_S_12_	289	33,268.58	8.76	68.93

**Table 2:** Germination indexes of each strain transgenic for *GmXTH-like26* at the germination stage.

Treatment	Line	Germination Rate (%)	Germination Vigor (%)	Germination Index
0 mM	WT	1 ± 0	0.65 ± 0.06	28.45 ± 1.41
	OE	1 ± 0	0.72 ± 0.06	30.76 ± 1.15
	KO	0.97 ± 0.02 *	0.6 ± 0.09	27.58 ± 1.55
100 mM	WT	0.63 ± 0.02	0.05 ± 0	5.46 ± 0.31
	OE	0.83 ± 0.04 **	0.23 ± 0.02 **	11.20 ± 0.28 **
	KO	0.47 ± 0.03 *	0 ± 0 **	3.75 ± 0.15 **
150 mM	WT	0.43 ± 0.07	0.02 ± 0.02	3.53 ± 0.39
	OE	0.63 ± 0.04 *	0.03 ± 0.02	5.62 ± 0.21 **
	KO	0.37 ± 0.04	0 ± 0	2.67 ± 0.18

* *p* < 0.05, ** *p* < 0.01.

**Table 3:** Seedling root indexes of each strain transfected with the *GmXTH-like26* gene.

Line	Treatment	Total Root Length (cm)	Root Volume (cm^3^)	Root Surface Area (cm^2^)	Average Diameter (mm)
WT	0 mM	14.65 ± 0.20	9.15 ± 0.11	39.54 ± 1.25	0.85 ± 0.11
	100 mM	9.27 ± 0.20	4.74 ± 0.22	28.66 ± 0.89	0.72 ± 0.05
	150 mM	4.61 ± 0.22	3.59 ± 0.15	17.42 ± 0.53	0.54 ± 0.03
OE	0 mM	15.56 ± 0.52 *	9.15 ± 0.11	41.10 ± 1.43	0.92 ± 0.13
	100 mM	10.03 ± 0.38	5.07 ± 0.08	30.57 ± 0.90	0.90 ± 0.09
	150 mM	5.51 ± 0.13 *	4.14 ± 0.06	19.47 ± 0.60 *	0.73 ± 0.08 *
KO	0 mM	14.13 ± 0.09	9.25 ± 0.06	36.13 ± 2.58	0.84 ± 0.09
	100 mM	8.20 ± 0.41	4.19 ± 0.22	20.48 ± 0.98 **	0.68 ± 0.06
	150 mM	3.73 ± 0.22 *	1.75 ± 0.23 **	14.67 ± 0.33 **	0.45 ± 0.03

* *p* < 0.05, ** *p* < 0.01.

## Data Availability

The original contributions presented in this study are included in the article material; further inquiries can be directed to the corresponding author.

## Supplementary Materials

The following supporting information can be downloaded at: https://www.mdpi.com/article/10.3390/plants15131948/s1, Figure S1: (A) Results of total RNA gel inspection; (B) PCR amplification of the GmXTH-like26 gene; (C) Sequence alignment results of the cloning vector pMD-18T-GmXTH-like26. Figure S2: (A) Plasmid double digestion test results; (B) Comparison of sequencing results. Figure S3: Genetic transformation process of soybean: (A) Seed germination; (B) Explant preculture; (C) Agrobacterium co-culture; (D,E) Resistance screening; (F) Adventitious bud elongation; (G) Root induction; (H) Regenerated seedling establishment. Figure S4: PCR detection of T_1_ generation positive soybean plants. (C) Electrophoresis results of Bar, 35S and NOS genes in overexpression lines; (D,E) Electrophoresis detection of Bar and Cas9 genes in gene-edited lines. M: DL 2000 DNA marker; P: positive control; 1: negative control; 2: wild-type JN18; 3–6: independent positive transgenic plants. Figure S5: PCR identification of T_2_ generation positive soybean plants. Figure S6: Bar rapid test strip detection of T_2_ generation soybean plants. Figure S7: Mutation target sequencing results of T_2_ generation gene-edited plants. Note: − = base deletion; S = base substitution; + = base insertion. Table S1: Names and sequences of all primers applied in this study. Table S2: Target site information of the gene-editing vector.
