# Supplementary material for: The Role of a Novel Gene, GmXTH-like26, in Improving Salt Tolerance in Soybean
Source: Plants (Basel). 2026 Jun 24;15(13):1948. doi: 10.3390/plants15131948 (PMC13364123; doi:10.3390/plants15131948)
Supplement: Supplementary file 1 [file plants-15-01948-s001.zip › plants-4329277-supplementary.pdf]

Supplementary Materials

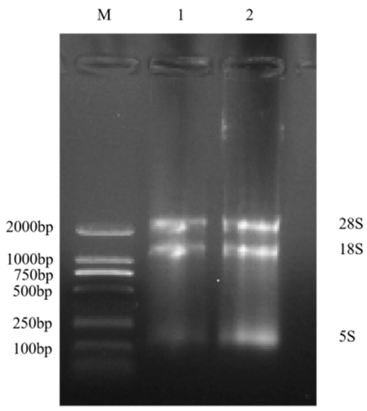

A

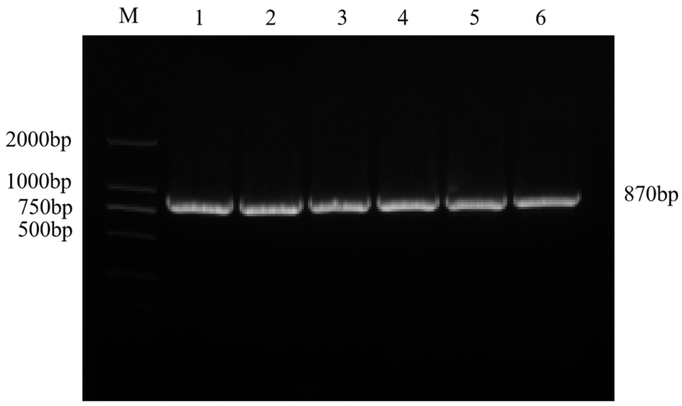

B

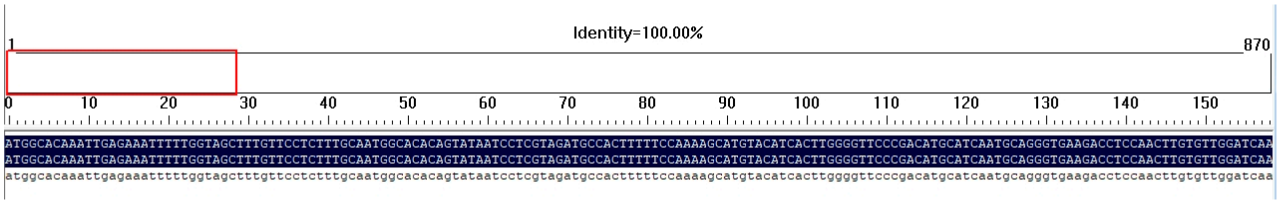

C

Fig.S1 A.Results of Total RNA gel Inspection (M: DL2000 molecular weight marker,1–2: RNA-derived PCR amplified products);B. PCR amplification of GmXTH-like26 gene(M: DL2000 molecular weight marker, 1–6: PCR amplified products); C.Sequence alignment results of cloning vector pMD-18T-GmXTH-like26

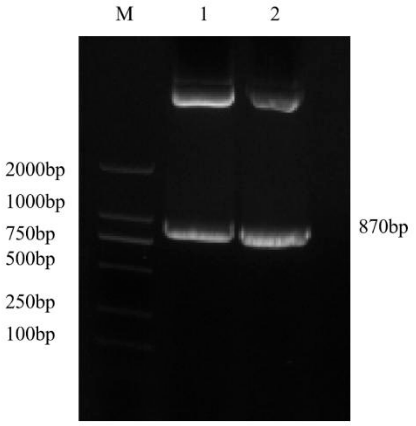

A

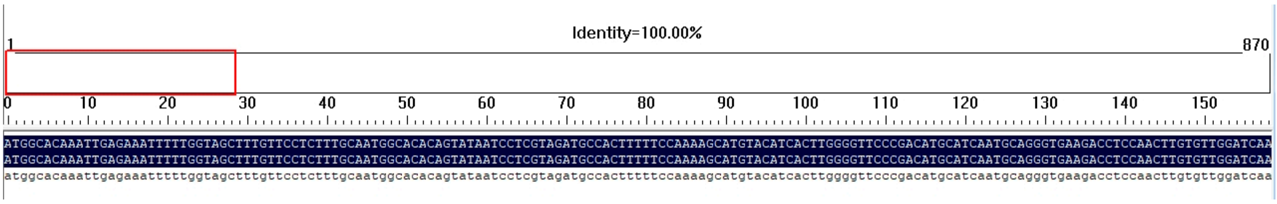

B

Fig. S2 A.Plasmid double digestion test results(M: DL2000 DNA Marker; 1–2: Restriction digestion results of recombinant plasmid); B.Comparison of sequencing results

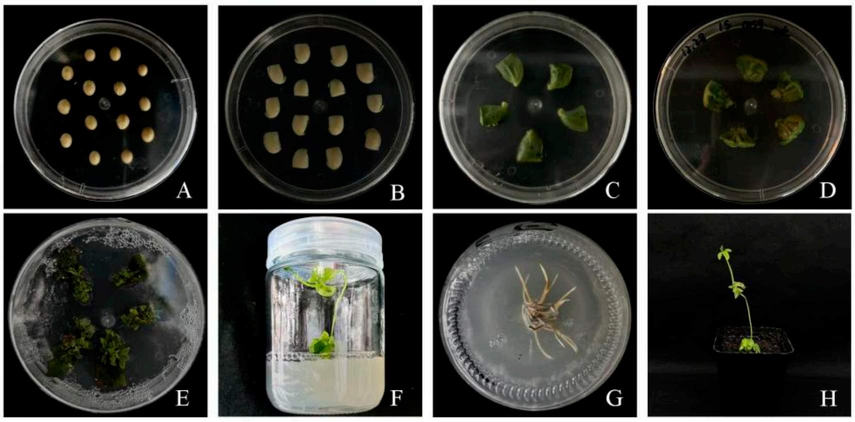

Fig. S3 Genetic transformation of soybean

A: germination; B: preculture; C: co-culture; D-E: screening; F: elongation; G: rooting; H: seedling establishment

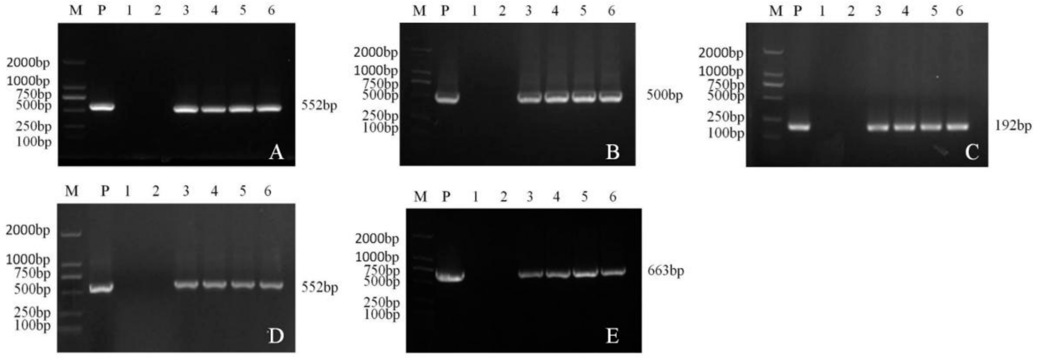

Fig.S4 PCR detection of T1 soybean positive plants

C: The electrophoresis detection results of Bar, 35S, and NOS genes in overexpressing plants; D-E: Bar and Cas9 gene electrophoresis detection results of gene edited plants(M: DL2000 DNA Marker; P: Positive control; 1: Negative control; 2: JN18; 3–6: Positive transgenic plants)

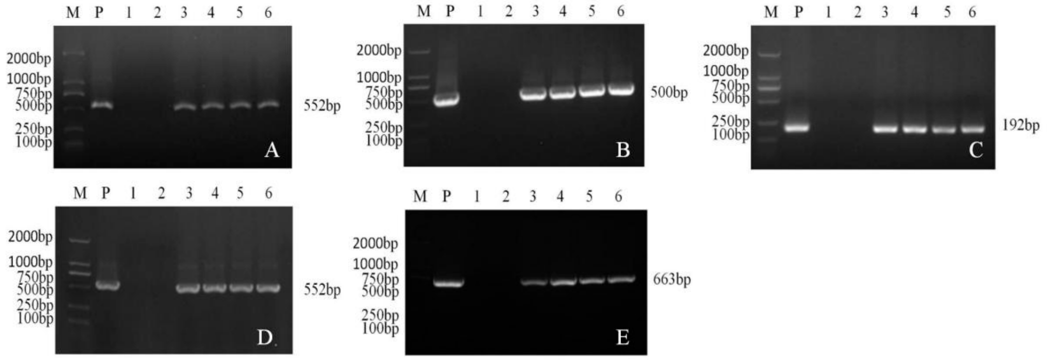

Fig.S5 PCR detection of T2 soybean positive plants(M: DL2000 DNA Marker; P: Positive control; 1: Negative control; 2: JN18; 3–6: Positive transgenic plants)

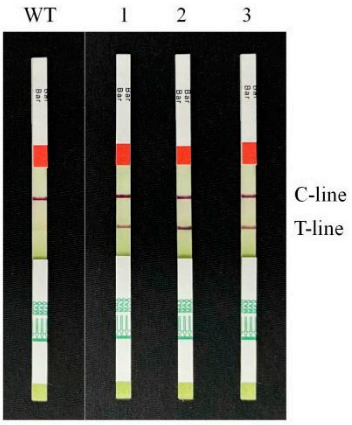

Fig .S6 The *Bar* test strip of T_2 generation plants

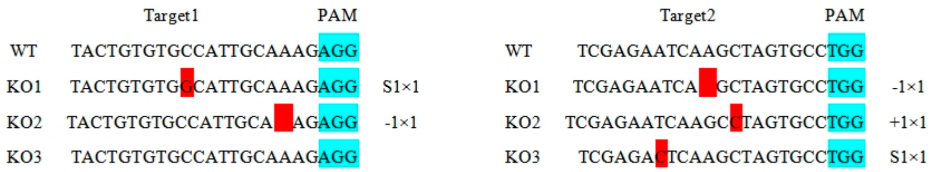

Fig.S7 Target detection results of T_2 generation gene editing plants

Note: - : base deletion; S: base replacement; + : base insertion

Tab S1 Names and sequences of primers used in this experiment

primer name	Sequence(5'-3')
GmXTH-like26-S	ATGGCACAAATTGAGAAATTTTTGGTA
GmXTH-like26-AS	TTAGAATTGTGACTTGAAGCATTCAG
pCAMBIA1302-GmXTH-like26-GFP-S	acgggggactcttgaccatggATGGCACAAATTGAGAAA TTTTTGGTA
pCAMBIA1302-GmXTH-like26-GFP-AS	tctcctttactagtcagatctTTAGAATTGTGACTTGAAGC ATTCAG
Bar-S	TCAAATCTCGGTGACGGGC
Bar-AS	ATGAGCCCAGAACGACGCC
35S-S	TAGAGGACCTAACAGAAC
35S-AS	CCGTGTTCTCTCCAAATG
NOS-S	GAATCCTGTTGCCGCTCTTG
NOS-AS	TTATCCTAGTTTGCGCGCTA
Cas9-S	CCCAAGAGGAACAGCGATAAG
Cas9-AS	GTCGATGGTGGTGTCAAAGT

Tab. S2 Gene editing vector target information

SG sequence	Target
GTGAACGGAATGTTGAACGG	TACTGTGTGCCATTGCAAAGAGG
GTGTTTGGAACCCTTGAGAG	TCGAGAATCAAGCTAGTGCCTGG
